# Large‐Area Fabrication of Hexaazatrinaphthylene‐Based 2D Metal‐Organic Framework Films for Flexible Photodetectors and Optoelectronic Synapses

**DOI:** 10.1002/advs.202305551

**Published:** 2024-01-23

**Authors:** Jiajun Song, Chun‐Ki Liu, Venkatesh Piradi, Changsheng Chen, Ye Zhu, Xunjin Zhu, Li Li, Wai‐Yeung Wong, Feng Yan

**Affiliations:** ^1^ Department of Applied Physics The Hong Kong Polytechnic University Hung Hom, Kowloon Hong Kong P. R. China; ^2^ Department of Chemistry Hong Kong Baptist University Kowloon Tong, Kowloon Hong Kong P. R. China; ^3^ School of Fashion and Textiles The Hong Kong Polytechnic University Hung Hom, Kowloon Hong Kong P. R. China; ^4^ Department of Applied Biology and Chemical Technology and Research Institute for Smart Energy The Hong Kong Polytechnic University Hung Hom, Kowloon Hong Kong P. R. China; ^5^ Research Institute of Intelligent Wearable Systems The Hong Kong Polytechnic University Hung Hom, Kowloon Hong Kong P. R. China

**Keywords:** 2D conjugated metal‐organic frameworks, flexible photodetectors, hexaazatrinaphthylene, large‐area thin films, optoelectronic synapses

## Abstract

2D conjugated metal‐organic frameworks (c‐MOFs) have emerged as promising materials for (opto)electronic applications due to their excellent charge transport properties originating from the unique layered‐stacked structures with extended in‐plane conjugation. The further advancement of MOF‐based (opto)electronics necessitates the development of novel 2D c‐MOF thin films with high quality. Cu‐HHHATN (HHHATN: hexahydroxyl‐hexaazatrinaphthylene) is a recently reported 2D c‐MOF featuring high in‐plane conjugation, strong interlayer π–π stacking, and multiple coordination sites, while the production of its thin‐film form has not yet been reported. Herein, large‐area Cu‐HHHATN thin films with preferential orientation, high uniformity, and smooth surfaces are realized by using a convenient layer‐by‐layer growth method. Flexible photodetectors are fabricated, showing broadband photoresponse ranging from UV to short‐wave infrared (370 to 1450 nm). The relatively long relaxation time of photocurrent, which arises from the trapping of photocarriers, renders the device's synaptic plasticity similar to that of biological synapses, promising its use in neuromorphic visual systems. This work demonstrates the great potential of Cu‐HHHATN thin films in flexible optoelectronic devices for various applications.

## Graphical abstract

High‐quality 2D conjugated metal‐organic framework Cu‐HHHATN (HHHATN: hexahydroxyl‐hexaazatrinaphthylene) thin films are fabricated and used for flexible optoelectronic devices. The photodetectors show broadband photoresponse ranging from UV to short‐wave IR. The trapping of photocarriers imparts the device with synaptic plasticity, promising its use in neuromorphic visual systems. This work demonstrates the great potential of Cu‐HHHATN in flexible optoelectronics.

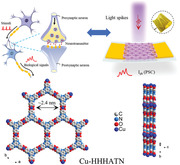

## 1 Introduction

Metal‐organic frameworks (MOFs), characterized by metal nodes interconnected with organic ligands to create ordered porous architectures, are noted for their utility in various applications such as separation,^[^ ^1^ ^]^ gas storage,^[^ ^2^ ^]^ catalysis,^[^ ^3^ ^]^ and sensor technologies.^[^ ^4^ ^]^ These applications mainly leverage the high porosity, large surface areas, and good tunability of MOFs. However, the majority of conventional MOFs suffer from poor charge transport properties which limit their applicability in (opto)electronic devices.^[^ ^5^ ^]^ Recently, 2D conjugated MOFs (c‐MOFs) have emerged as promising materials for (opto)electronic applications.^[^ ^6^, ^7^, ^8^, ^9^, ^10^ ^]^ They not only retain the notable features of conventional MOFs but also demonstrate excellent electrical transport properties due to their unique layered‐stacked structures with extended in‐plane conjugation. Recent advancements in material design and thin film preparation methods have expanded the use of 2D c‐MOFs in diverse (opto)electronic devices including field‐effect transistors (FETs),^[^ ^11^, ^12^, ^13^ ^]^ electrochemical transistors,^[^ ^8^, ^14^ ^]^ supercapacitors,^[^ ^15^ ^]^ spintronics,^[^ ^16^, ^17^, ^18^ ^]^ solar cells,^[^ ^9^ ^]^ photodetectors (PDs),^[^ ^7^, ^19^ ^]^ and artificial synapses.^[^ ^20^ ^]^ Nevertheless, only a limited number of high‐quality 2D c‐MOF thin films are available due to the challenges in their preparation,^[^ ^21^ ^]^ necessitating the development of novel 2D c‐MOF films with high quality for (opto)electronic devices.

To enrich the 2D c‐MOFs family, efforts have been devoted to the design of organic ligands.^[^ ^10^, ^22^, ^23^, ^24^ ^]^ Currently, organic ligands (e.g., benzene and triphenylene) containing a single class of chelating functional groups (e.g., ─OH, ─SH, and ─NH_2_) are frequently used for constructing 2D c‐MOFs, while the ligands with heteroatoms are rarely reported.^[^ ^10^, ^23^ ^]^ The incorporation of heteroatoms in organic ligands can offer additional coordination sites, potentially imbuing the resulting 2D c‐MOFs with unique properties for novel applications. Recently, a novel ligand, hexahydroxyl‐hexaazatrinaphthylene (HHHATN) containing two types of coordination sites has been synthesized for the production of 2D c‐MOFs.^[^ ^23^ ^]^ 2D c‐MOF Cu‐HHHATN powders have been successfully prepared and used for the electroreduction of CO_2_. It was found that the favorable π–π stacking of the HHHATN ligand contributed to the high stability of the 2D c‐MOF and the exposed nitrogen sites demonstrated high affinity to CO_2_. Such 2D c‐MOF with large heteroatoms‐incorporated ligand cores is expected to exhibit good charge transport properties due to the high in‐plane conjugation and strong interlayer π–π stacking. On the other hand, the phenanthroline units can offer additional coordination sites for guest binding, offering a potential way for tuning the material properties via host‐guest chemistry.^[^ ^25^, ^26^ ^]^ These features render the Cu‐HHHATN a promising candidate for thin‐film (opto)electronic devices. However, as of yet, the production of Cu‐HHHATN thin films has not been reported.

Herein, large‐area fabrication of 2D c‐MOF Cu‐HHHATN thin films is demonstrated for the first time. High‐quality Cu‐HHHATN films featuring preferential orientation, high uniformity, and smooth surfaces are successfully prepared by using a convenient layer‐by‐layer growth method. The optical bandgap of the semiconducting Cu‐HHHATN is estimated at 1.46 eV, and the energy levels are determined according to the ultraviolet photoelectron spectroscopy (UPS) and absorption spectra. Flexible PDs based on Cu‐HHHATN thin films are developed, showing broadband photoresponse ranging from UV to short‐wavelength infrared (SWIR) (370 to 1450 nm). The extended photoresponse to 1450 nm with photon energy less than the bandgap can be attributed to the hopping of photocarriers between trap states. The relatively long relaxation time of photocurrent, originating from the trapping of photocarriers, inspires us to use the device as a flexible optoelectronic synapse. The synaptic device demonstrates various plasticity similar to that of biological synapses and shows potential in complicated imaging processing. This work demonstrates the great potential of the novel Cu‐HHHATN thin film in flexible optoelectronic devices.

## 2 Results and Discussion

**Figure 1:**
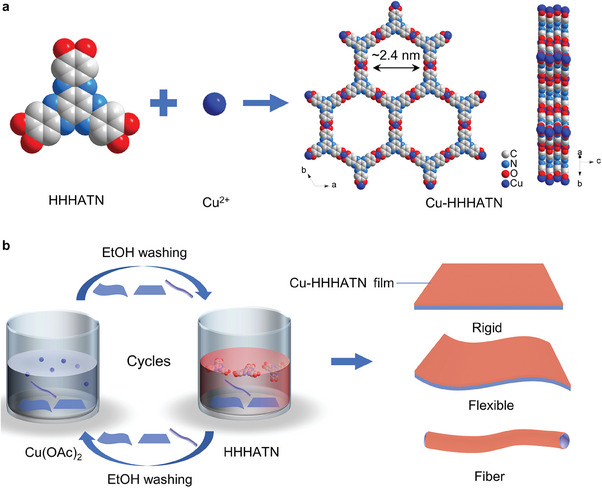
a) Synthetic route and crystalline structure of Cu‐HHHATN. Hydrogen atoms are hidden for clarity. b) Preparation procedure of Cu‐HHHATN thin films on various substrates.

To obtain Cu‐HHHATN thin films, we first synthesized the HHHATN ligand using the synthetic route shown in Scheme S1, Supporting Information (see Supporting Information for experimental details).^[^ ^23^ ^]^ The successful synthesis of the HHHATN ligand was confirmed via the nuclear magnetic resonance (NMR) spectra (Figure S1, Supporting Information). Then, Cu‐HHHATN thin films were prepared through a layer‐by‐layer self‐assembling growth method, involving a reaction between HHHATN ligands and Cu^2+^ ions (**Figure** **1**a). Figure 1b delineates the procedure of the layer‐by‐layer growth of Cu‐HHHATN thin films. The detailed experimental procedure is included in the Supporting Information. Briefly, the process begins with the functionalization of a substrate using hydroxyl groups. Then, the substrate is submerged in an ethanolic solution of Cu(OAc)_2_ to anchor Cu^2+^ ions onto the hydroxyl groups. Post immersion, the substrate is meticulously washed with ethanol to avoid excess Cu^2+^ ions. Then, the substrate is immersed in an ethanolic solution of HHHATN ligands, allowing the hydroxyl groups of HHHATN to bond with Cu^2+^ ions, forming a continuous 2D c‐MOF thin layer. After a subsequent washing stage with ethanol, the process is repeated until the desired thickness is attained. Notably, this convenient solution processing method allows the in situ growth of Cu‐HHHATN films on various substrates including rigid, flexible, and fibrous substrates. The formation of Cu‐HHHATN on the substrate was initially examined by Fourier transform infrared (FT‐IR) spectra (Figure S2, Supporting Information), in which the broad peak at ≈3400 cm^−1^ of HHHATN disappeared after the growth of Cu‐HHHATN, indicating the completion of the reaction after the layer‐by‐layer growth process.

**Figure 2:**
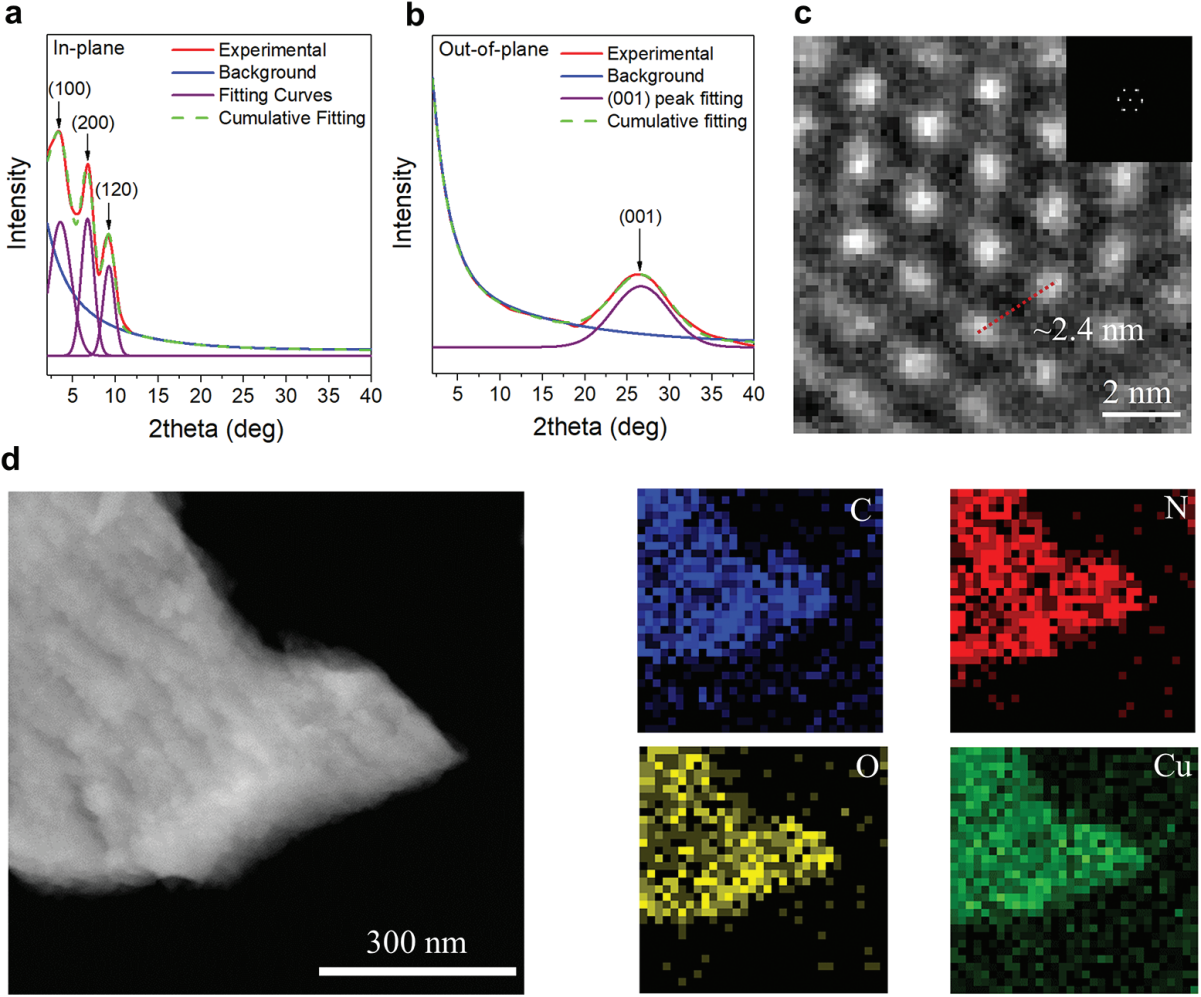
a) In‐plane and b) out‐of‐plane GIXRD patterns of a Cu‐HHHATN thin film. c) HRTEM image of the Cu‐HHHATN thin film. Inset: FFT of the area. d) Scanning transmission electron microscopy (STEM) image of the Cu‐HHHATN thin film alongside its EDX elemental mapping with respect to C, N, O, and Cu.

Grazing incidence X‐ray diffraction (GIXRD) measurements were performed to investigate the crystallinity and orientation of the Cu‐HHHATN film. **Figures** **2**a,b show the in‐plane and out‐of‐plane GIXRD spectra of a Cu‐HHHATN film, respectively. All the measured peaks are in close agreement with the simulated powder XRD spectrum (Figure S3, Supporting Information). The in‐plane GIXRD pattern reveals three peaks at 2*θ* = 3.5°, 6.8°, and 9.2°, corresponding to the (100), (200), and (120) planes, respectively, while the out‐of‐plane GIXRD pattern exhibits a single peak (2*θ* = 27°) corresponding to the (001) plane. The notable discrepancy between the in‐plane and out‐of‐plane GIXRD patterns indicates that the Cu‐HHHATN crystals grow orientationally along the [001] axis on the substrate. The relatively broad XRD peaks are attributable to the small crystal size, a characteristic previously reported for 2D c‐MOF thin films.^[^ ^7^, ^17^, ^27^ ^]^ Gaussian functions aptly fit all the XRD peaks. The crystal size and microstrain in the Cu‐HHHATN thin film were estimated to be 5.7 nm and 6.7%, respectively, by using the Williamson–Hall (W–H) method ( Figure S4, Supporting Information).^[^ ^28^ ^]^ The relatively large microstrain, compared to inorganic thin films, can be attributed to the abundant soft organic ligands present in the Cu‐HHHATN film. These values resemble those of previously reported Cu_3_(HHTT)_2_ and are reasonable for 2D c‐MOF thin films.^[^ ^7^ ^]^ High‐resolution transmission electron microscopy (HRTEM) was used to characterize the Cu‐HHHATN thin film detached from the substrate. The HRTEM image (Figure 2c) and the corresponding fast Fourier transform (FFT) pattern clearly indicate the hexagonal nanoporous structure of Cu‐HHHATN with a pore size of ≈2.4 nm. Furthermore, the energy dispersive X‐ray (EDX) mapping (Figure 2d) verifies the uniform distribution of C, N, O, and Cu atoms across the entire film, affirming the chemical composition of Cu‐HHHATN.

The scanning electron microscopy (SEM) images (Figure S5a–c, Supporting Information) of Cu‐HHHATN films with various thicknesses indicate the formation of continuous films, devoid of any pinholes, entirely covering the substrates. This is crucial for carrier transport in (opto)electronic devices. Atomic force microscopy (AFM) was employed to investigate the topography of the thin films. As depicted in Figure S5d–f, Supporting Information, the root mean square surface roughness of Cu‐HHHATN films with various thicknesses (25, 54, and 82 nm, Figure S5g‐i, Supporting Information) are 1.2, 2.6, and 3.9 nm, respectively. These values are less than 5% of the overall film thickness, signifying the good smoothness of the films. The uniformity of a large‐area Cu‐HHHATN film (60 mm × 60 mm) was evaluated using AFM. The AFM images (Figure S6, Supporting Information) obtained from various regions of the film exhibited consistent roughness values and revealed a smooth surface, indicating a high degree of uniformity across the entire large‐area film. A series of characterizations confirm the successful production of high‐quality Cu‐HHHATN films with preferential orientation, high uniformity, and smooth surfaces, demonstrating the potential for optoelectronic applications.

**Figure 3:**
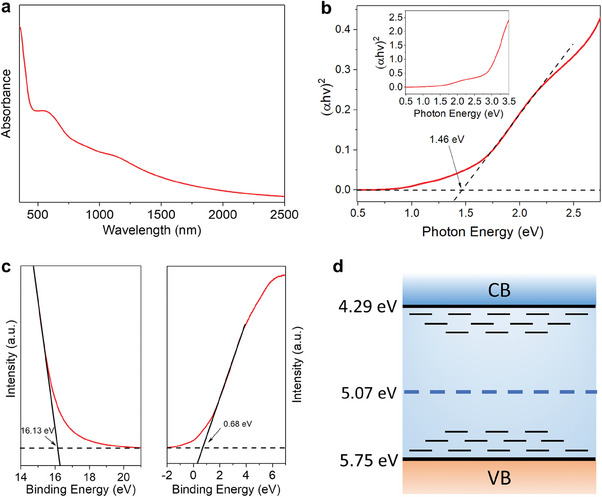
a) Absorption spectra and b) the corresponding Tauc plot of a Cu‐HHHATN thin film. c) UPS spectra of the Cu‐HHHATN thin film. d) Band diagram of Cu‐HHHATN. Energy levels in the bandgap correspond to tail states.

Next, the semiconducting properties of the Cu‐HHHATN film were investigated. **Figures** **3**a,b show the absorption spectrum and the corresponding Tauc plot of a Cu‐HHHATN thin film, respectively. It is notable that the absorption tail extends to the SWIR range, while the optical band gap is estimated to be ≈1.46 eV, lying within the visible light range. The extended absorption range can be attributed to the electronic transition from the valence band to gap states or from gap states to the conduction band. UPS was employed to study the energy levels of Cu‐HHHATN. As indicated in the secondary cut‐off region of the UPS spectrum (Figure 3c), the binding energy of Cu‐HHHATN is 16.13 eV. Under a UV light source of 21.20 eV, the Fermi energy is calculated to be 5.07 eV. On the other hand, the energy difference from the Fermi level to the valence band maximum (VBM) is 0.68 eV, as revealed in the valence band region of the UPS spectrum. Combining the results from UPS and absorption spectra, the band diagram of Cu‐HHHATN is determined, as shown in Figure 3d.

To measure the carrier mobility of the Cu‐HHHATN, we fabricated a top‐gated FET based on Cu‐HHHATN thin films by successively depositing a high‐*k* dielectric poly(vinylidene fluoride‐trifluoroethylene‐chlorofluoroethylene) (P(VDF‐TrFE‐CFE)) (56/36.5/7.5 mol%) terpolymer (thickness: ≈600 nm) and an aluminum gate electrode on top of the 2D c‐MOF film (Figure S7a, Supporting Information). The transistor shows a typical ambipolar behavior, in which the channel conductivity can be modulated effectively via field‐effect doping (Figure S7b, Supporting Information). According to the transfer characteristic, the hole and electron mobilities are estimated to be ≈9.1 × 10^−4^ and ≈6.9 × 10^−4^ cm^2^ V^−1^ s^−1^, respectively. The relatively low carrier mobilities obtained from the device can be attributed to a high density of trap states in the 2D c‐MOF channel. An ambipolar performance can be observed in a transistor based on a narrow bandgap semiconductor, while the optical bandgap energy of the Cu‐HHHATN characterized by light absorption is around 1.46 eV, which is even higher than that of Si (i.e., 1.12 eV). So, the ambipolar behavior can be attributed to the tail states of the conduction band and valence band. The energy difference between the two types of tail states gives an effective bandgap energy much lower than the optical bandgap energy, which is consistent with the extended absorption tail of the Cu‐HHHATN film far beyond the absorption edge. To the best of our knowledge, this is the first demonstration of a top‐gate FET with a 2D c‐MOF thin film as the channel material.

**Figure 4:**
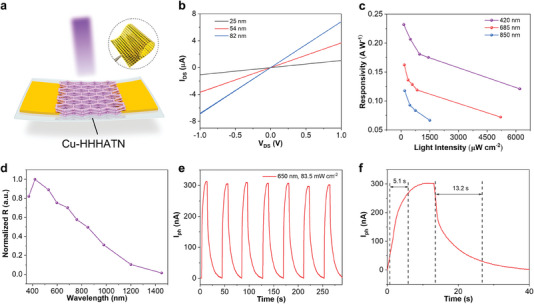
a) Diagram of a Cu‐HHHATN‐based flexible PD. Inset: Photograph of a flexible device array. b) Dark *I–V* curves of PDs with various film thicknesses (25, 54, and 82 nm). c) Dependence of responsivity on light intensity for three different wavelengths (film thickness: 82 nm). d) Normalized responsivity of the device at different wavelengths ranging from 370 to 1450 nm. e) Transient photocurrent response of the device under continuous light pulses. f) Enlarged view of the photocurrent response under one light on–off cycle, showing the rise and fall time.

PDs are an important class of optoelectronic devices, underpinning diverse imaging and sensing applications.^[^ ^29^, ^30^, ^31^, ^32^, ^33^, ^34^ ^]^ Low‐cost flexible PDs with a broadband spectral response are highly desirable for emerging wearable and implantable applications.^[^ ^30^, ^35^, ^36^, ^37^, ^38^ ^]^ Considering their broad absorption spectra, facile processing, intrinsic flexibility, and tunability, Cu‐HHHATN thin films were employed to construct flexible PDs. A typical photoconductor based on Cu‐HHHATN is fabricated on an ultrathin polyimide (PI) substrate (**Figure** **4**a, see Supporting Information for experimental details). Benefiting from the convenient layer‐by‐layer growth of Cu‐HHHATN films, a flexible device array on a large‐area substrate can be easily realized, as inserted in Figure 4a. Figure 4b shows the dark *I–V* curves of the devices with various film thicknesses, indicating the ohmic contact between the Cu‐HHHATN films and the gold (Au) electrodes, which is important for photocarrier transport. It is reasonable to find that the channel conductivity increases with film thickness. Under light illumination, the photons with sufficient energy can excite electrons near the valence band and generate electron–hole pairs in the Cu‐HHHATN. When a bias voltage is applied between the electrodes, these photocarriers will be collected in respective electrodes, contributing to photocurrents (*I*_ph_). Responsivity (*R*) is a key figure of merit of a PD that quantifies the strength of photoresponse, which is given by

(1) $$R\;=\frac{I_{\mathrm{ph}}}{WLE_{e}}\;$$

where *W*, *L*, and *E*_e_ are the channel width, channel length, and incident light intensity, respectively. Figure 4c shows the relationship between the responsivity and intensity of incident light at three wavelengths, indicating a maximum responsivity of 0.23 A W^−1^ under 420‐nm illumination. The responsivity of the device is nearly two orders of magnitude higher than that (≤4 mA W^−1^) of previously reported broadband MOF‐based photodetectors.^[^ ^7^, ^19^ ^]^ Notably, the responsivity decreases with increasing light intensity for all wavelengths, which can be attributed to the saturation of photocurrent under a high light intensity. Under high light intensity, a large number of photocarriers are accumulated in the Cu‐HHHATN film, which significantly increases the recombination possibility and reduces the carrier lifetime, resulting in low responsivity. The uniformity of a 5 × 5 flexible array (Figure S8a, Supporting Information) was tested under illumination, revealing consistent photocurrent responses across all devices (Figure S8b, Supporting Information). To evaluate the mechanical stability of the flexible device, the device was subjected to 800 bending cycles against a glass rod with a radius of 2 mm. The photocurrent was measured after every 200 bending cycles. As depicted in Figure S8c, Supporting Information, the device exhibited good stability, with minimal degradation of less than 10% in photocurrent after 800 bending cycles.

Spectral response describes the sensitivity of a PD to the incident light at different wavelengths, determining the application scenarios of the device. As shown in Figure 4d, the response spans from UV (370 nm) to SWIR (1450 nm), indicating a broadband photoresponse of the device. It is worth noting that the spectral response is highly dependent on the absorption spectrum (Figure 3a). The optical band gap of Cu‐HHHATN is measured to be 1.46 eV, corresponding to a wavelength of 850 nm, while the extended photoresponse from 850 to 1450 nm can be attributed to the hopping of photocarriers between gap states, similar to another 2D c‐MOF film under the illumination of infrared light.^[^ ^7^ ^]^

To characterize the transient response, continuous light pulses were applied to illuminate the device, and the photocurrent response was monitored in real‐time. As shown in Figure 4e, the device demonstrated stable and reproducible switching under multiple light on‐off cycles. The rise and fall time, defined as the time duration between 10% and 90% of the maximum photocurrent, are measured to be 5.1 and 13.2 s, respectively, as shown in Figure 4f. The falling edge can be fitted well with a double exponential function^[^ ^37^ ^]^

(2) $$I_{\mathrm{ph}}=A_{f1}\;\exp\left(-\frac{t}{\tau_{f1}}\right)\;\;+\;A_{f2}\exp\left(-\frac{t}{\tau_{f2}}\right)$$

where *A*_f1_ and *A*_f2_ are magnitudes for the two falling parts, and *τ*_f1_ and *τ*_f2_ are time constants. The time constants *τ*_f1_ and *τ*_f2_ are extracted as 0.69 and 7.4 s, respectively, as shown in Figure S9, Supporting Information. The fast decay could be attributed to the carrier recombination between the conduction band and valance band, while the prolonged recombination of trapped carriers can lead to a long relaxation time. Such transient behaviors have been previously observed in PDs based on low‐dimensional semiconductors with similar band structures.^[^ ^39^ ^]^

**Figure 5:**
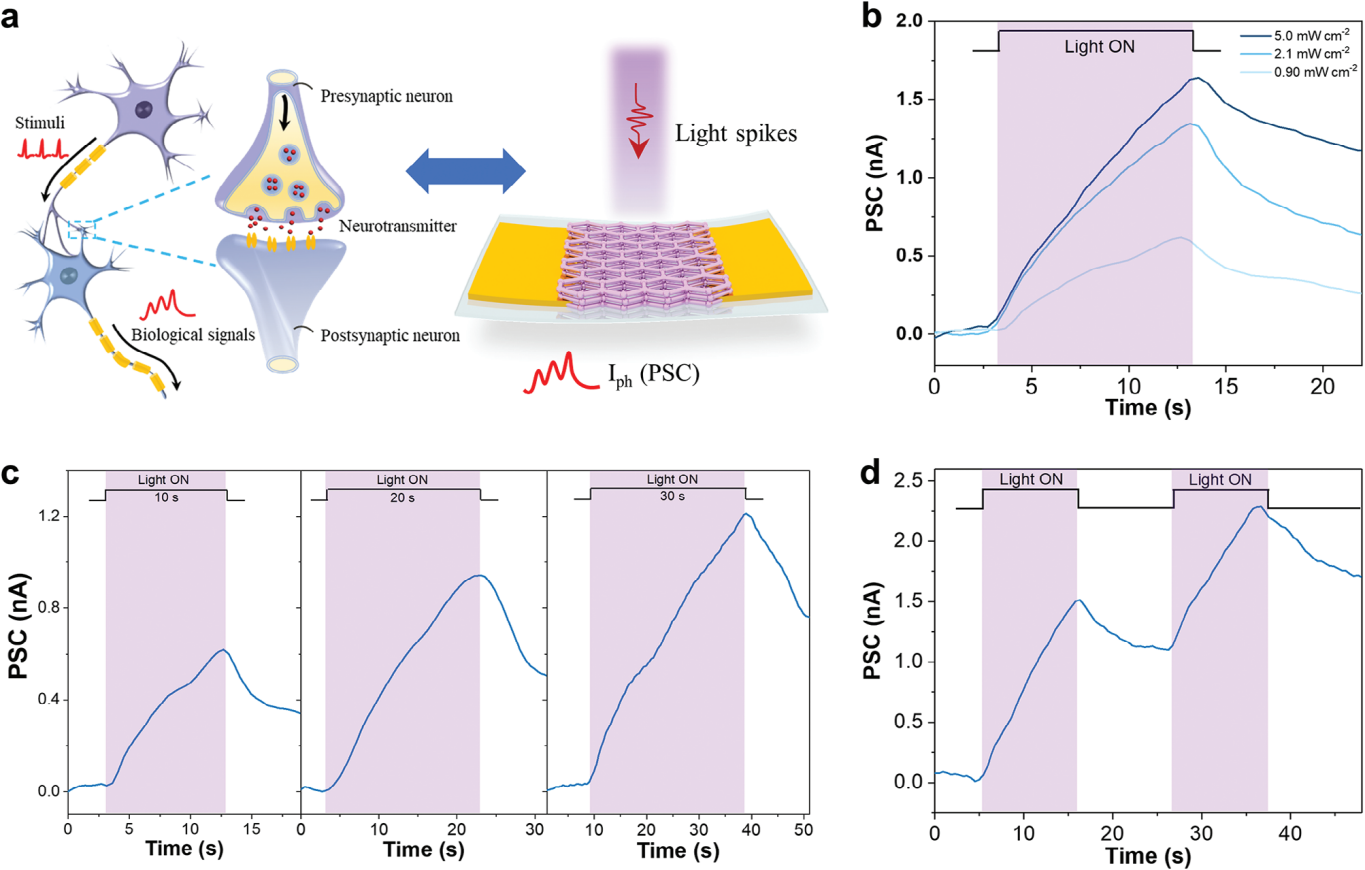
a) Diagram of a biological synapse and the schematic structure of a flexible optoelectronic synapse based on Cu‐HHHATN. b) Post‐synaptic currents of the optoelectronic synapse under light spikes with different light intensities. c) Post‐synaptic currents of the device under light stimuli with different illumination duration times. d) Post‐synaptic current under a light pulse pair with a 10‐s interval.

The relatively long decay time originating from the trapping of photocarriers inspired us to use the device as a flexible optoelectronic synapse that can find potential applications in neuromorphic visual systems.^[^ ^40^, ^41^, ^42^, ^43^, ^44^, ^45^ ^]^ In a biological system, the neural signals triggered by external stimuli such as optical information are communicated between neurons through a synapse, where neurotransmitters are passed from a presynaptic neuron to a postsynaptic neuron and then modulates the synapse weight (**Figure** **5**a). Similarly, in our optoelectronic synapse, light spikes can trigger a post‐synaptic current (PSC) in the form of a photocurrent response. Figure 5b presents the PSC of a Cu‐HHHATN‐based synapse under light spikes with various light intensities (wavelength: 420 nm; pulse duration: 10 s). The PSC increased under a light stimulus and then gradually decayed after the light was off, which is similar to the short‐term plasticity (STP) of biological synapses. The synaptic strength including PSC and relaxation time was enhanced by increasing light intensity due to the increased number of photogenerated carriers. Similar STP characteristics were observed across a broad range of wavelengths, spanning from UV to IR (Figure S10, Supporting Information). The PSC exhibited variations due to the distinctive responsivities of the device at different wavelengths. Thus, our device demonstrates the capacity for capturing a wide range of optical stimuli beyond visible light. Besides light intensity, light dosage (illumination duration time) can tune the synaptic strength of the device. As shown in Figure 5c, increasing the width of optical pulses (wavelength:420 nm; intensity: 0.90 mW cm^−2^) led to a more significant PSC response. Thus, the Cu‐HHHATN‐based synapse can sense not only light intensity but also light dosage, demonstrating potential implementations in complicated image processing.^[^ ^40^ ^]^ Paired‐pulse facilitation (PPF) is a typical phenomenon of STP in an excitatory synapse, in which the PSC increases under a paired spike.^[^ ^46^ ^]^ This behavior was emulated by applying two consecutive presynaptic light spikes (wavelength:420 nm; pulse duration: 10 s; intensity: 5.0 mW cm^−2^) on the device. It can be observed that the PSC triggered by the second light pulse is significantly higher than that triggered by the first one (Figure 5d), which can be attributed to the accumulated photocarriers trapped in the 2D c‐MOF film. The PPF index, defined as the ratio of the second PSC to the first PSC, was calculated to be 151% with a time interval of 10 s. Figure S11, Supporting Information, shows the PPF index at different time intervals (5, 15, and 20 s). Notably, the PPF index gradually decreased as the time interval increased, exhibiting characteristics similar to those observed in biological synapses. We also found that the synaptic strength of the device can be augmented by increasing the number of spikes. Figure S12, Supporting Information, demonstrates that an increase in pulse number leads to an enhanced PSC response, resembling the plasticity exhibited by biological synapses.

## 3 Conclusion

In conclusion, large‐area 2D c‐MOF Cu‐HHHATN thin films featuring preferential orientation, high uniformity, and smooth surfaces are realized for the first time by using a convenient layer‐by‐layer growth method. The optical bandgap of the semiconducting Cu‐HHHATN film is estimated at 1.46 eV, and the band diagram is confirmed according to UPS and absorption spectra. A top‐gated FET based on Cu‐HHHATN thin film is fabricated to measure the carrier mobility, exhibiting a typical ambipolar behavior due to the conduction process through tail states. Flexible PDs based on Cu‐HHHATN are constructed, showing broadband photoresponse ranging from UV to SWIR. The extended photoresponse to 1450 nm with photon energy less than the optical bandgap can be attributed to the hopping of photocarriers between gap states. The relatively long relaxation time observed in the PDs, resulting from the trapping of photocarriers by defects motivates the utilization of the device as a flexible optoelectronic synapse. The synaptic device demonstrates various plasticity akin to that of biological synapses and shows potential for use in neuromorphic visual systems. The novel Cu‐HHHATN thin film offers promising opportunities for the development of advanced flexible (opto)electronic devices with multiple functions.

## Conflict of Interest

The authors declare no conflict of interest.

## Supporting information

Supporting Information

## Data Availability

The data that support the findings of this study are available from the corresponding author upon reasonable request.
