# Supplementary material for: Large‐Area Fabrication of Hexaazatrinaphthylene‐Based 2D Metal‐Organic Framework Films for Flexible Photodetectors and Optoelectronic Synapses
Source: Adv Sci (Weinh). 2024 Jan 23;11(13):2305551. doi: 10.1002/advs.202305551 (PMC10987135; doi:10.1002/advs.202305551)
Supplement: Supplementary file 1 — Supporting Information [file ADVS-11-2305551-s001.pdf]

ADVANCED SCIENCE
Open Access

Supporting Information

for *Adv. Sci.*, DOI 10.1002/advs.202305551

Large-Area Fabrication of Hexaazatrinaphthylene-Based 2D Metal-Organic Framework Films for Flexible Photodetectors and Optoelectronic Synapses

Jiajun Song, Chun-Ki Liu, Venkatesh Piradi, Changsheng Chen, Ye Zhu, Xunjin Zhu, Li Li*, Wai-Yeung Wong* and Feng Yan**

Supporting Information

Large-Area Fabrication of Hexaazatrinaphthylene-Based 2D Metal–Organic Framework Films for Flexible Photodetectors and Optoelectronic Synapses

Jiajun Song, Chun-Ki Liu, Venkatesh Piradi, Changsheng Chen, Ye Zhu, Xunjin Zhu, Li Li,* Wai-Yeung Wong,* and Feng Yan**

Experimental Procedures

1. Synthesis Procedure of HHHATN Ligand

Synthesis of Compound 1[1]

The 65% of concentrated nitric acid (100 mL) was taken in a 250 mL round bottom flask, and cooled to 0 ^oC under ice bath. 1,2 Dimethoxybenzene (10 mL) was added carefully into the above solvent under vigorous stirring. Then the reaction mixture was allowed to warm to room temperature, which was heated for 5 h at 80 ^oC. After cooling down to room temperature, the yellow precipitate was formed. The crude product was filtered, and washed with deionized water for several times until the filtrate was neutral, then dried to afford compound 1. Yield: 17.31 g (88%). ^1H NMR (400 MHz, DMSO-*d*6) δ (ppm): 7.76 (s, 2H), 3.96 (s, 6H).

Synthesis of Compound 2[1]

A mixture of compound 1 (2.0 g, 8.76 mmol) and Pd/C catalyst was placed in a 100 mL reaction flask and 40 mL of ethanol was added under nitrogen. The mixture was cooled to 0 ^oC by using ice water bath, then hydrazine monohydrate (4.26 mL, 87.6 mmol) was carefully added dropwise. The resulting mixture was refluxed for 3 h at 80 ^oC. After reaction completed, the mixture was cooled, filtered and washed with hot ethanol for several times. The resultant solid was concentrated and dried under vacuum for overnight to afford white crystalline compound 2. Yield: 1.16 g (74%). ^1H NMR (400 MHz, $CDCl_3$) δ (ppm): 6.37 (s, 2H), 3.79 (s, 6H), 3.19 (s, 4H).

Synthesis of Compound 3[1]

A mixture of compound 2 (1.5 g, 8.92 mmol) and hexaketocyclohexane octahydrate (0.43 g, 2.55 mmol) was taken in a three-neck 100 mL round bottom flask and kept under nitrogen condition. Glacial acetic acid (20 mL) was added to the mixture slowly, then it was refluxed for 24 h. After reaction completed, the mixture was cooled to room temperature and filtered, and washed with hot water for several times. The filtrate was dried in vacuum to obtain an orange-yellow product 3. Yield: 0.62 g (1.09 mmol, 38%). ^1H NMR (400 MHz, $CDCl_3$) δ (ppm): 7.90 (s, 6H), 4.18 (s, 18H). MALDI-TOF (m/z) compound 3 ($C_{30}H_{24}N_6O_6$), calculated 564.5580, found 565.1015.

Synthesis of the final Compound Hexahydroxyl-hexaazatrinaphthylene (HHHATN)[1]

The compound 3 (300 mg, 0.53 mmol) was taken in a three-neck 100 mL round bottom flask and kept under nitrogen condition. Then anhydrous dichloromethane (DCM) was added, and cooled to 0 ^oC by using an ice water bath. After that, 3.2 mL of 1.0 M boron tribromide (BBr_3) solution in anhydrous dichloromethane (3.2 mmol) was injected carefully. The reaction mixture was stirred for 3 days at room temperature. Then the reaction was quenched by adding water (100 mL) slowly, and washed with hot water to give the dark solid as the desired compound HHHATN. Yield: 0.2 g (0.42 mmol, 74%). ^1H NMR (400 MHz, DMSO-*d*6) δ (ppm): 10.99 (s, 6H), 7.59 (s, 6H). ^{13}C NMR (100 MHz, DMSO-*d*6) δ (ppm): 153.3, 140.8, 140.4, 109.5. MALDI-TOF (m/z) compound HHHATN ($C_{24}H_{13}N_6O_6$), calculated 481.0852, found 481.5741.

2. Material Characterizations

HHHATN Ligand: ^1H NMR and ^{13}C NMR spectra were recorded on Bruker AC 300 P using the deuterated solvents ($CDCl_3$, and DMSO-*d*6) with the tetramethylsilane as the internal reference. High-resolution MALDI-TOF mass spectra were obtained with a Bruker Autoflex

MALDI-TOF mass spectrometer. Fourier transform infrared spectroscopy (FTIR) was performed on a Spectrum 100 (Perkin Elmer, Inc., USA) spectrometer with a scan range of 4000-400 cm^{-1}.

Cu-HHHATN Thin Film: The out-of-plane and in-plane XRD spectra was obtained by X-Ray diffraction (XRD), Rigaku SmartLab X-Ray diffractometer. SEM images were observed under a field emission scanning electron microscope (FESEM) (Tescan MAIA3). AFM images were recorded using scanning probe microscope (Bruker NanoScope 8). The thicknesses of the MOF films were characterized by a surface profiler (Bruker DektakXT). The HRTEM image was taken using a field emission electron microscope (JEM-2100F). The absorption spectra were recorded with a LAMBDA 1050+ UV/Vis/NIR spectrophotometer (PerkinElmer). UPS spectra was recorded using an X-Ray Photoelectron Spectrometer System (Thermo Fisher Nexsa).

The grain size and macrostrain of the Cu-HHHATN are estimated according to W-H method, which assumes uniform strain in all crystalline directions and is used extensively for polycrystalline materials.[2] In this model, the peak broadening or full width at half maximum (FWHM) for each XRD peak can be expressed as:

$$\beta_{hkl} = \left(\frac{k\lambda}{Dcos\theta}\right) + 4\varepsilon tan\theta, \quad (1)$$

where k, D and ε are shape factor, crystal size and microstrain, respectively. Rearranging the equation yields the following:

$$\beta_{hkl} cos\theta = \frac{k\lambda}{D} + 4\varepsilon sin\theta \quad (2)$$

A W-H plot is obtained by plotting $\beta_{hkl} cos\theta$ against $4sin\theta$ (Figure S4). The value of k is assigned to be 0.94, which is a general value originally derived by Scherrer and used widely for thin films.[3-5] From linear fitting of the data, crystal size and microstrain can be estimated from the y-intercept and the slope.

3. Device Fabrication

Pre-patterned Substrate Preparation: For photodetectors and optoelectronic synapses, flexible PI substrates were used. PI substrates were first cleaned by ultrasonication bath using deionized water, acetone, and IPA, followed by drying the substrate with stream of nitrogen gas. Next, 10nm-Cr/100nm-Au electrodes were deposited by magnetron sputtering after the desired area was patterned by standard photolithography process. The channel length and width of the device used in this work are 5 μm and 800 μm, respectively.

For top-gated FETs, glass substrates were used instead. The bottom and top electrodes are prepared by thermal evaporation through shadow masks. The bottom electrodes are 10nm-Cr/100nm-Au and the thickness of the top Al electrode is ~80 nm. The channel length and width of the devices are 100 μm and 2000 μm, respectively.

Fabrication of Cu-HHHATN Thin Film: The above patterned substrates were first functionalized using oxygen plasma for 30 minutes. Next, the substrates were used for preparing Cu-HHHATN films according to previous report.[6, 7] The functionalized substrates were immersed alternatively into 1.0 mM copper acetate ethanolic solution and 0.1 mM HHHATN ethanolic solution for 20 minutes and 40 minutes, respectively (Figure 1b). The substrates were washed with pure ethanol to remove excessive precursor between each immersion step. The film thickness can be controlled by cycle numbers. The process is stopped after a desired thickness is achieved.

Fabrication of Dielectric Layer on Cu-HHHATN Thin Film: To fabricate P(VDF-TrFE-CFE) thin films, P(VDF-TrFE-CFE) (56/36.5/7.5 mol%) powder was dissolved into methyl ethyl ketone (MEK) at a concentration of 70 mg ml^{-1} and spin-coated onto the Cu-HHHATN thin film with a speed of 3000 rpm.

4. Device Characterizations

The electrical and optoelectrical measurements for the devices were performed by a semiconductor parameter analyzer (Keithley 4200). The light sources are LEDs with different wavelengths. The transient response of the device was measured by applying light pulses. For the characterizations of synaptic plasticity, light pulses with different patterns were applied to stimulate the device.

Schemes and Figures

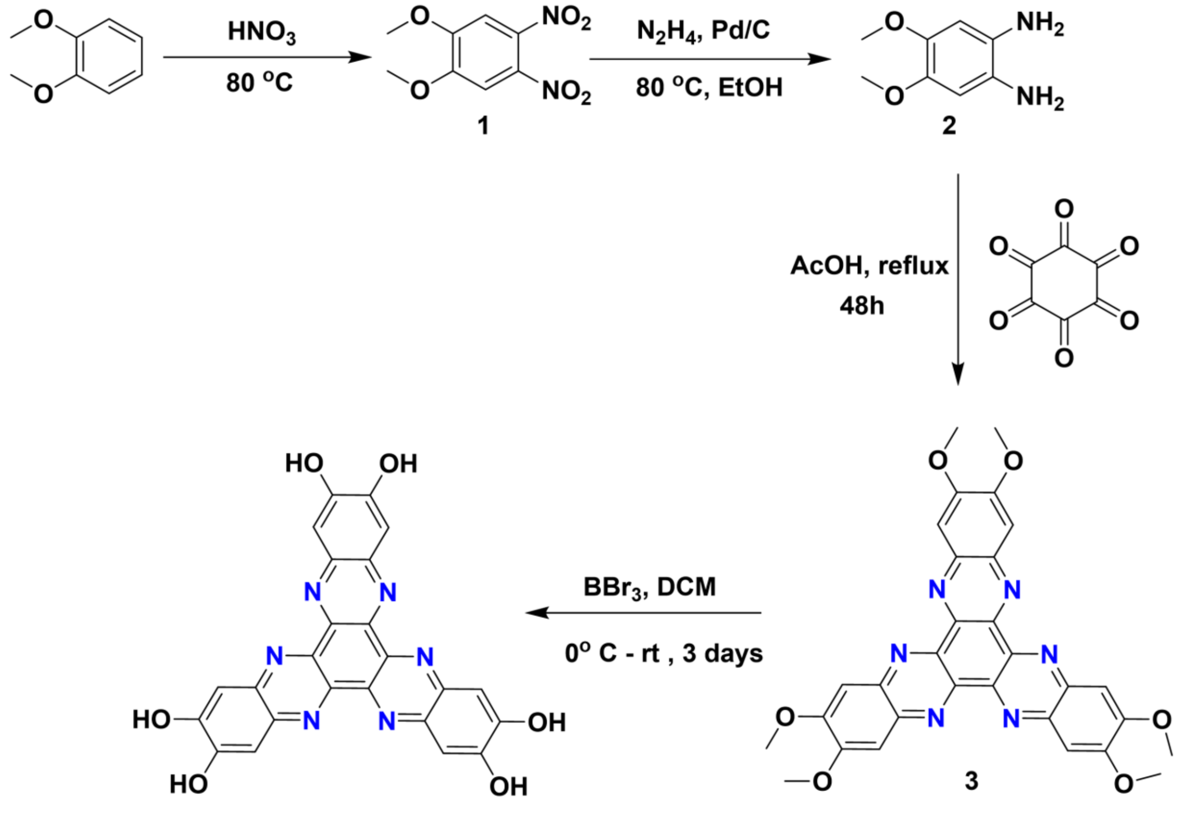

Hexahydroxyl-hexaazatrinaphthylene (HHHATN)

Scheme S1. Synthetic route of HHHATN ligand.

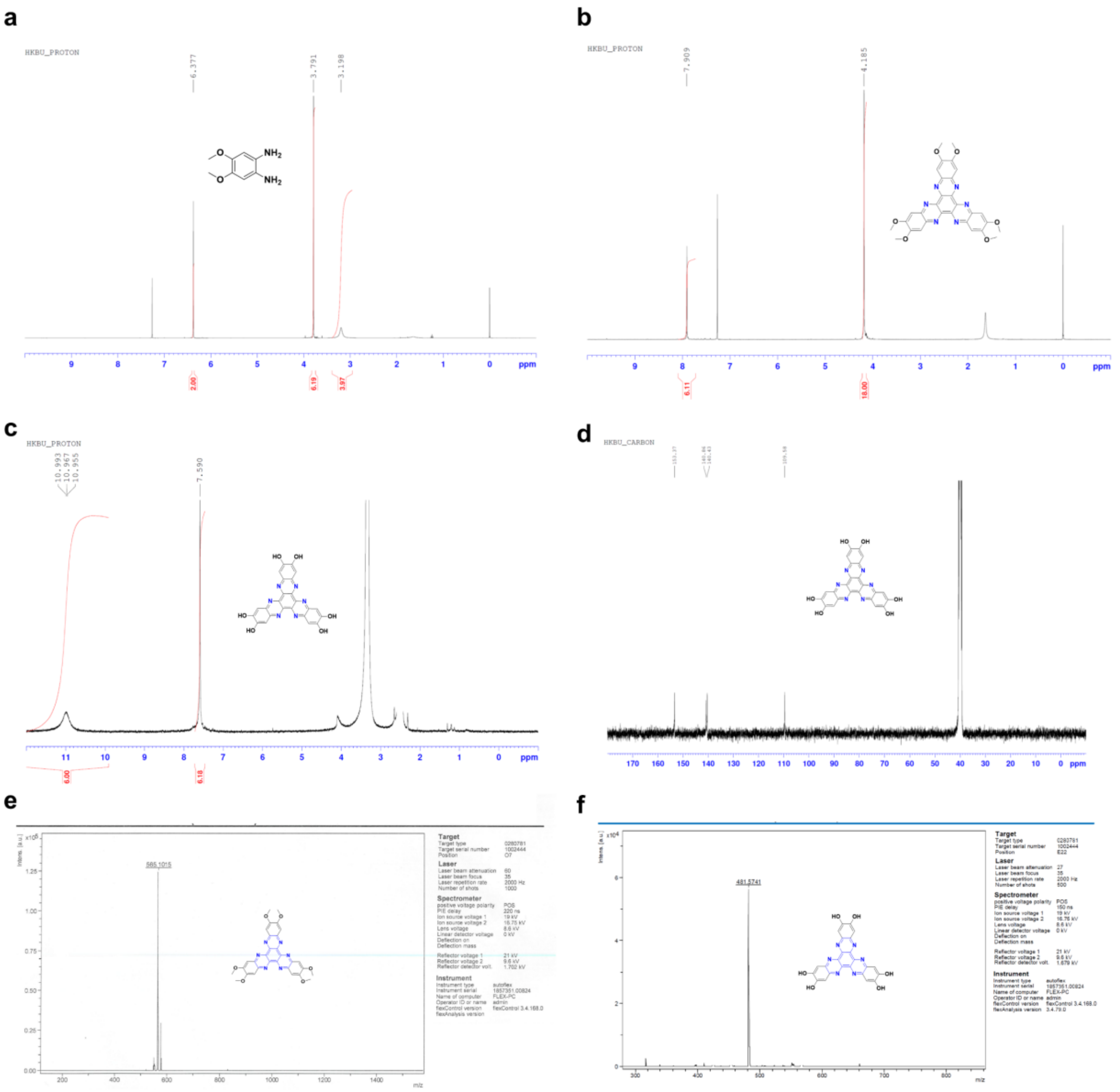

Figure S1. a) ^{1}H NMR of compound 2 recorded in $CDCl_3$. b) ^{1}H NMR of compound 3 recorded in $CDCl_3$. c) ^{1}H NMR of compound HHHATN recorded in DMSO-d_6. d) ^{13}C NMR of compound HHHATN recorded in DMSO-d_6. High-resolution MALDI-TOF mass spectra of e) compound 3 and f) HHHATN.

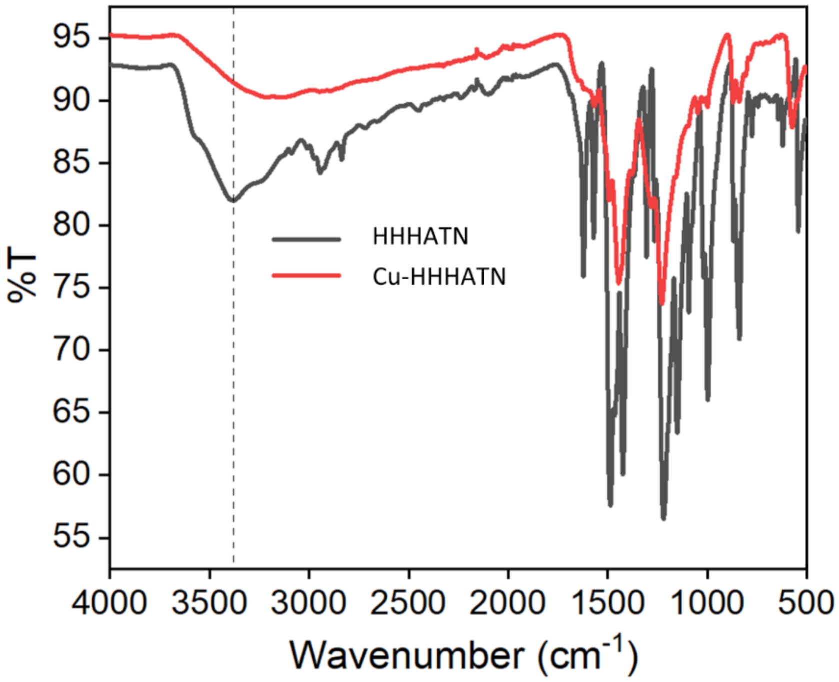

Figure S2. FTIR spectra of HHHATN and Cu-HHHATN.

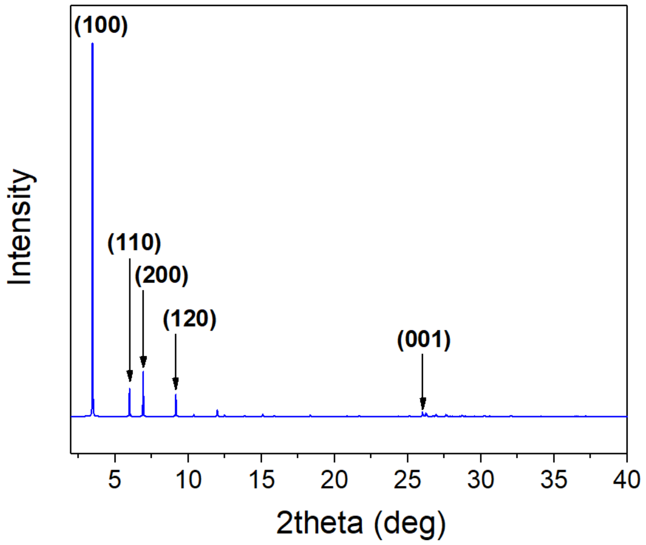

Figure S3. Simulated PXRD spectrum of Cu-HHHATN.

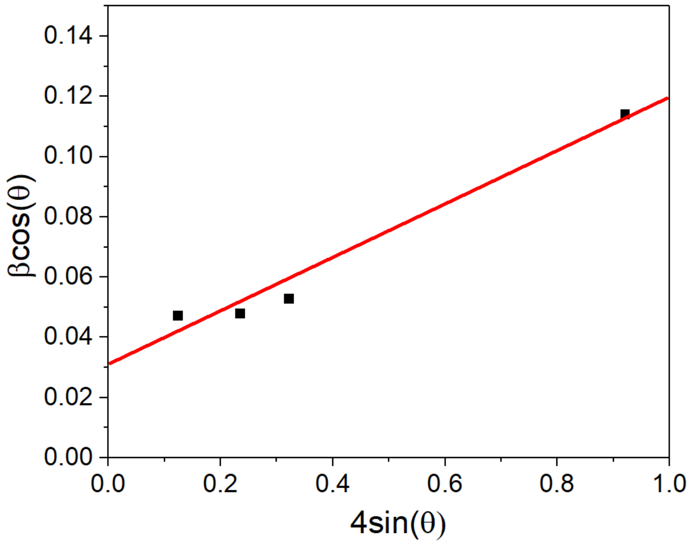

Figure S4. W-H plot corresponding to the GIXRD patterns shown in Figure 2a, b.

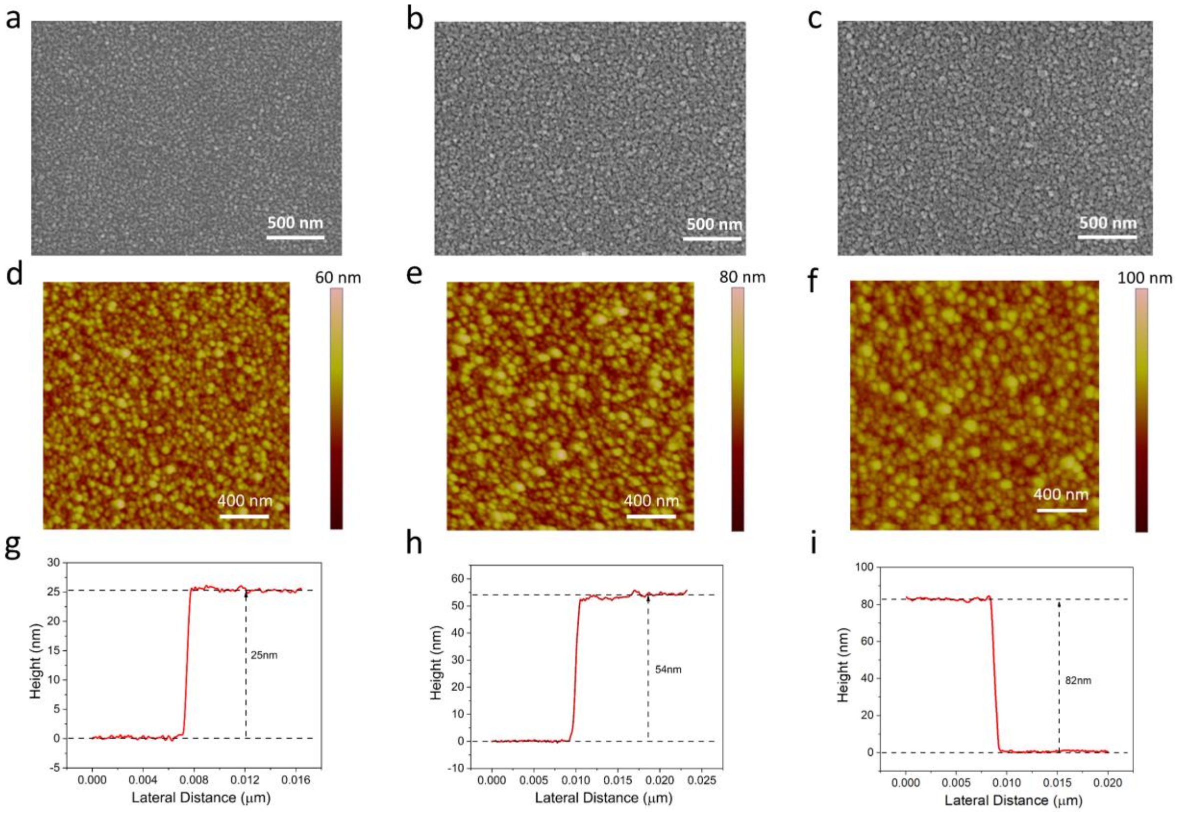

Figure S5. a-c) SEM images, d-f) AFM images and g-i) thicknesses of Cu-HHHATN thin films with 4, 8 and 12 growth cycles, respectively.

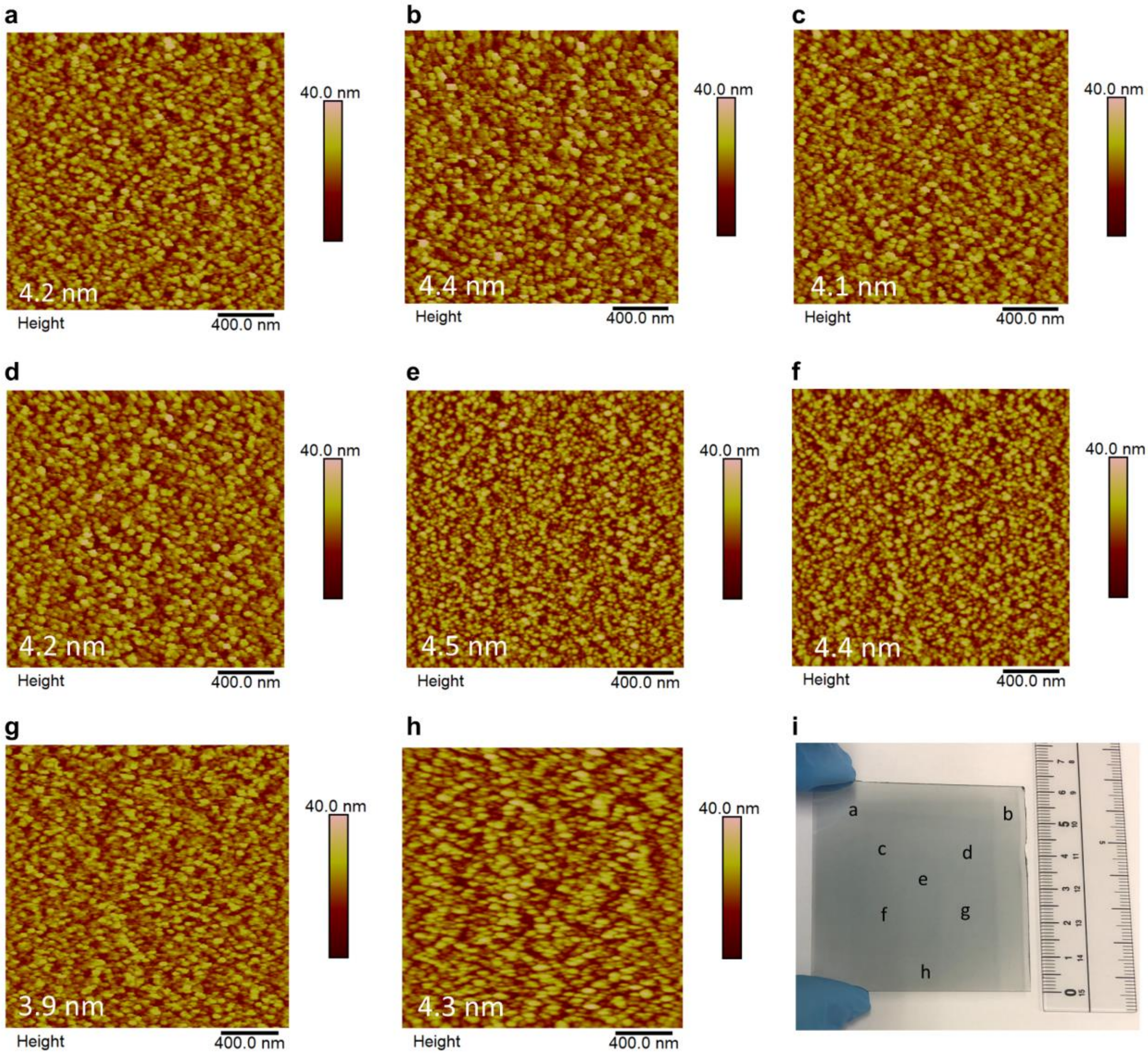

Figure S6. (a-h) AFM images obtained from various regions of a 12-cycle Cu-HHHATN film deposited on a large-area substrate (60 mm × 60 mm), with the RMS roughness values indicated in the bottom-left corners of each image. (i) Photograph of the large-area film, indicating the specific positions where the corresponding AFM images (a-h) were acquired.

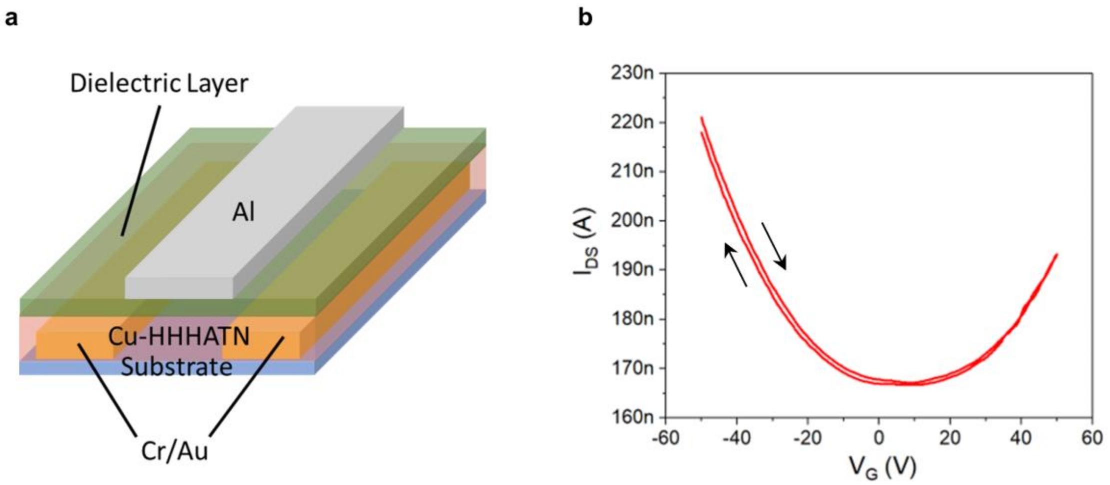

Figure S7. a) Device structure of a top-gate FET based on Cu-HHHATN. b) Transfer curve of the device (V_{DS} = 1.0 V).

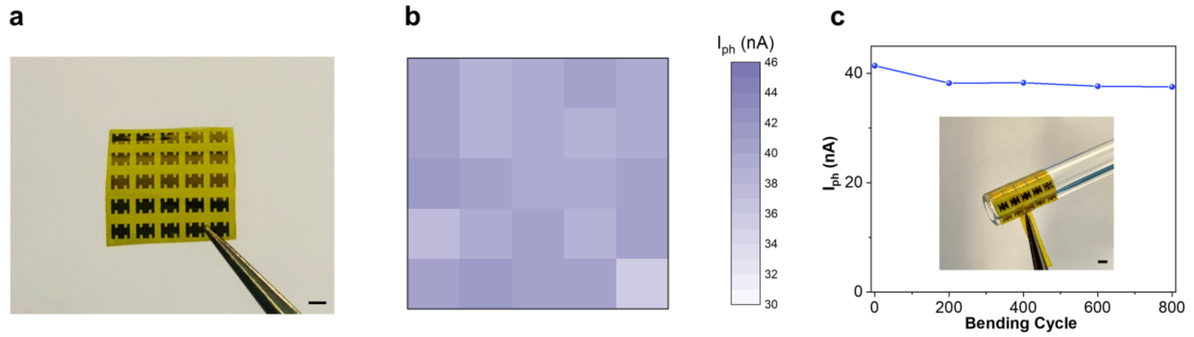

Figure S8. a) Photograph of a 5 x 5 flexible array. Scale bar: 1.0 mm. b) Photocurrent distribution of the devices on the array under light illumination (Wavelength:420 nm; intensity: 9.6 mW cm^{-2}). c) Photocurrent variation of a device under successive bending tests. Insert: Photograph of the array subjected to bending tests. Scale bar: 1.0 mm.

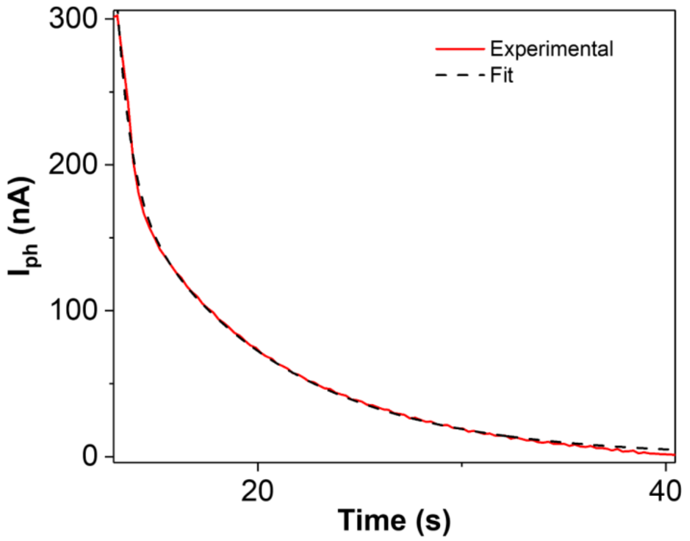

Figure S9. Fitting of the decay edge in Figure 4f with a double exponential function.

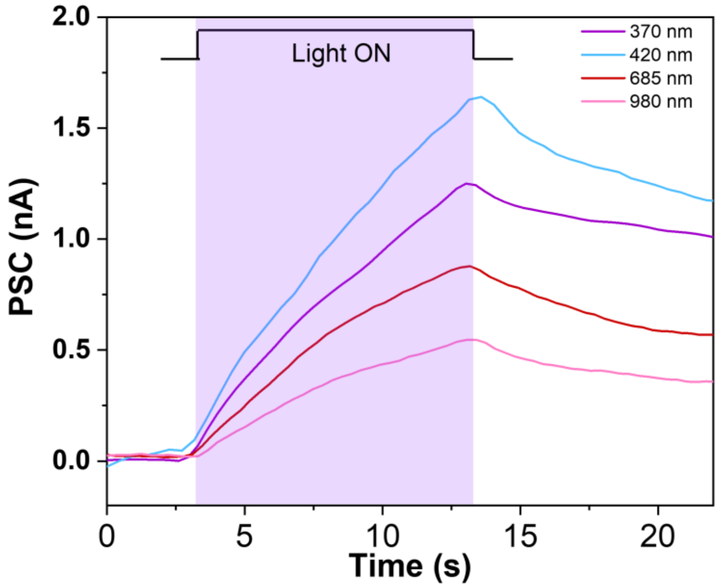

Figure S10. Post-synaptic currents of the optoelectronic synapse under light spikes with different light wavelengths (Intensity: 5.0 mW cm^{-2}).

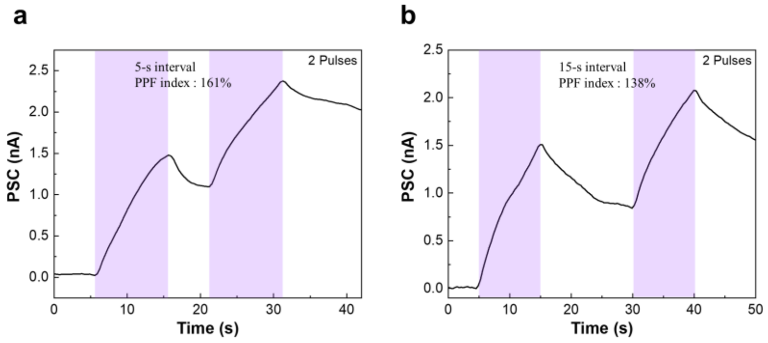

Figure S11. a) Post-synaptic current under a light pulse pair with a 5-s interval. b) Post-synaptic current under a light pulse pair with a 15-s interval. c) Post-synaptic current under a light pulse pair with a 20-s interval.

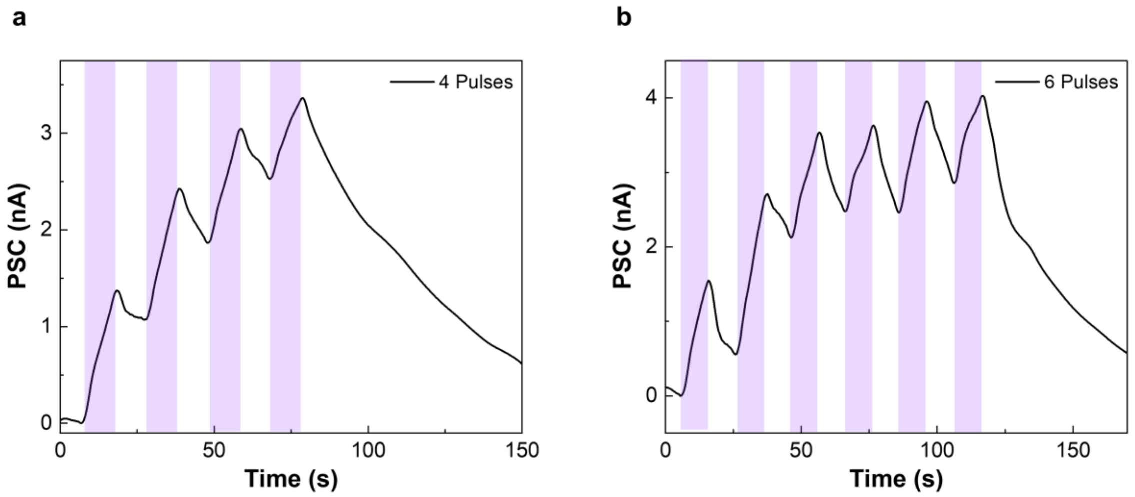

Figure S12. a) Post-synaptic current under 4 light pulses. b) Post-synaptic current under 6 light pulses.

[1] Y. Liu, S. Li, L. Dai, J. Li, J. Lv, Z. Zhu, A. Yin, P. Li, B. Wang, *Angew. Chem., Int. Ed.* **2021**, *60*, 16409-16415.
[2] V. Mote, Y. Purushotham, B. Dole, *Journal of theoretical and applied physics* **2012**, *6*, 1-8.
[3] H. Kafashan, *J. Electron. Mater.* **2019**, *48*, 1294-1309.
[4] D. J. Lim, N. A. Marks, M. R. Rowles, *Carbon* **2020**, *162*, 475-480.
[5] P. Scherrer, *Nachr. Ges. Wiss. Goettingen, Math.-Phys. Kl* **1918**, *2*, 98–100.
[6] J. Song, H. Liu, Z. Zhao, X. Guo, C. K. Liu, S. Griggs, A. Marks, Y. Zhu, H. K. Law, I. McCulloch, F. Yan, *Sci. Adv.* **2023**, *9*, eadd9627.
[7] C. K. Liu, V. Piradi, J. Song, Z. Wang, L. W. Wong, E. H. Tan, J. Zhao, X. Zhu, F. Yan, *Adv. Mater.* **2022**, *34*, e2204140.
